# Efficacy of different sequential patterns after crizotinib progression in advanced anaplastic lymphoma kinase‐positive non–small cell lung cancer

**DOI:** 10.1111/1759-7714.14455

**Published:** 2022-05-13

**Authors:** Xiya Ma, Shaoxing Yang, Kun Zhang, Jing Xu, Panpan Lv, Hongjun Gao, Haifeng Qin, Hong Wang, Xiaoqing Liu

**Affiliations:** ^1^ Academy of Military Medical Sciences, Academy of Military Sciences Beijing China; ^2^ Department of Oncology The Fifth Medical Center of Chinese PLA General Hospital Beijing China; ^3^ Medical School of Chinese PLA Beijing China

**Keywords:** anaplastic lymphoma kinase, crizotinib, non–small cell lung cancer, progression, sequential

## Abstract

**Background:**

The efficacy difference between the second‐ and third‐generation of anaplastic lymphoma kinase‐tyrosine kinase inhibitors (ALK‐TKIs) after crizotinib failure in advanced *ALK*‐positive non–small cell lung cancer (NSCLC) has not been clarified. This study evaluates the efficacy of different sequential patterns after crizotinib progression.

**Methods:**

Data of patients who met the study criteria were retrospectively analyzed. The Kaplan–Meier method was used to draw survival curves, log‐rank method was used to compare the differences between groups, and Cox multivariate analysis was used to evaluate the significance of influencing factors.

**Results:**

A total of 128 patients developed disease progression after crizotinib. The overall survival (OS) of 57 patients in the sequential second‐generation ALK‐TKIs group was significantly longer than that of 65 patients with other systemic treatment (58.5 months vs. 33.0 months, *p* < 0.001); The OS of the direct sequential lorlatinib group was significantly longer than the second‐generation ALK‐TKIs group (114.0 months vs. 58.5 months, *p* = 0.020). Similarly, of the 48 patients who developed disease progression after first‐ and second‐generation ALK‐TKIs treatment, 16 patients with sequential lorlatinib had significantly longer OS than the others (62.0 months vs. 43.0 months, *p* = 0.014). The progression‐free survival (PFS) of second‐line and third‐ or later‐line lorlatinib were statistically different (20.0 months vs. 5.5 months, *p* = 0.011).

**Conclusions:**

The application of next‐generation ALK‐TKIs after crizotinib progression significantly prolonged survival, whereas direct sequencing lorlatinib seemed advantageous. Similarly, lorlatinib also prolonged survival in patients with first‐ and second‐generation ALK‐TKIs failure.

## Graphical abstract

In this retrospective analysis of 128 patients with crizotinib failure, we found that the application of next‐generation ALK‐TKIs after crizotinib progression significantly prolonged survival, whereas direct sequencing lorlatinib seemed advantageous. In addition, lorlatinib also prolonged survival in patients with first‐ and second‐generation ALK‐TKIs failure.

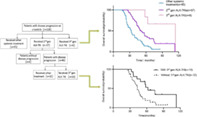

## INTRODUCTION

Lung cancer is associated with high morbidity and mortality, and non–small cell lung cancer (NSCLC) accounts for about 80%–90% of cases of lung cancer. Anaplastic lymphoma kinase (*ALK)* gene rearrangement was identified in NSCLC in 2007^1^; the application of tyrosine kinase inhibitors (TKIs) targeting *ALK* fusion mutation developed rapidly and is proven to have good efficacy and safety in various clinical trials.

Although TKIs demonstrated good tumor response, drug resistance during treatment was a concomitant problem. Despite the good efficacy of crizotinib, disease progression is inevitable at 10.9–12.7 months.^2^, ^3^, ^4^, ^5^, ^6^ The most common mechanism of drug resistance is acquired point mutation in the *ALK* gene. The second‐generation ALK‐TKIs, ceritinib, alectinib, and brigatinib, have gradually become the standard treatment after the development of resistance to crizotinib and are even administered as first‐line treatment.^7^, ^8^, ^9^ The third‐generation inhibitor lorlatinib is also proven to overcome the resistance to other ALK‐TKIs.^10^ As more next‐generation ALK‐TKIs are being developed and marketed, more options are available for *ALK*‐positive NSCLC patients in China. Therefore, it is essential to clarify the various drug resistance mechanisms and accordingly choose ALK‐TKIs for each case.^11^, ^12^

Further, effective sequential administration of ALK‐TKIs to maximize patient survival is a challenge for clinicians. Currently, there are few large clinical trials comparing the efficacy of different next‐generation ALK‐TKIs after crizotinib resistance. The survival outcomes of patients with advanced *ALK*‐positive NSCLC following administration of different sequential patterns need to be supported by more real‐world data. Therefore, we mainly evaluated the efficacy of sequential *ALK* inhibitors after crizotinib resistance in clinical practice and analyzed the influence of clinical characteristics and different sequential patterns on overall survival (OS).

## METHODS

### Patients

Patients with *ALK*‐positive locally advanced or metastatic NSCLC with disease progression undergoing crizotinib treatment at the Lung Oncology Department of the Fifth Medical Center of The People's Liberation Army (PLA) General Hospital from 2011 to 2019 were screened. Accepted test methods for molecular profiling were fluorescence in situ hybridization (FISH), immunohistochemistry (IHC), real time‐polymerase chain reaction (RT‐PCR), or next generation sequencing (NGS). This study was approved by the Ethics Committee of the Fifth Medical Center of the General Hospital of Chinese People's Liberation Army and was conducted according to the principles of the Declaration of Helsinki. Because this retrospective study did not harm the rights and health of patients, and protected their privacy and personal information, the ethics committee waived the requirement for informed consent.

### Efficacy evaluation

The efficacy evaluation of all enrolled patients was based on the response evaluation criteria in solid tumors (RECIST) version 1.1. The objective response rate (ORR) is the percentage of complete response (CR) and partial response (PR) in evaluable cases. The disease control rate (DCR) is the percentage of cases with response (CR + PR) and stable disease (SD) in evaluable cases. Progression‐free survival (PFS) is defined as the time from the start of treatment with an *ALK* inhibitor to the onset of disease progression or death from any cause. OS was defined as the time from the start of treatment with an *ALK* inhibitor to death from any cause. The last follow‐up was on October 31, 2021.

### Statistical analysis

Descriptive statistics were used to summarize patients' demographic and baseline clinical characteristics. The Kaplan–Meier method was used to draw survival curves. The log‐rank method was used to conduct univariate analysis on the differences in OS between groups. Variables with *p* < 0.05 and clinical significance were included in Cox multivariate analysis. All statistical analyses were performed using Statistical Package for the Social Sciences (SPSS) 25.0 (IBM). All tests were double tailed when *p* < 0.05 was considered statistically significant.

## RESULTS

### Clinical characteristics

**FIGURE 1:**
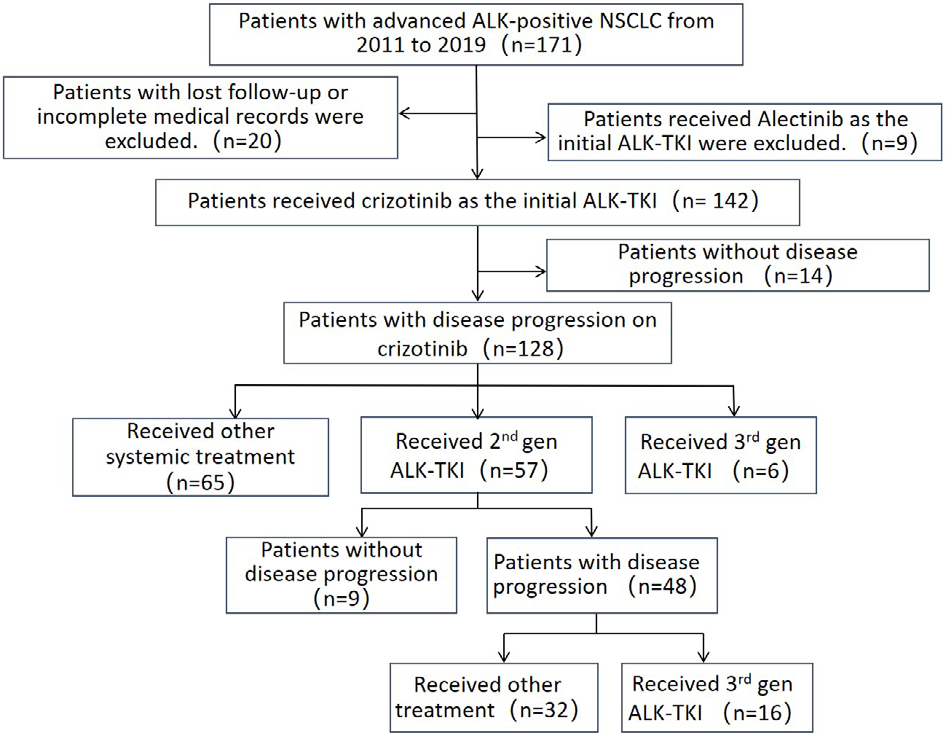
Patient flowchart.

There were 171 patients with *ALK*‐positive advanced NSCLC admitted to our department from 2011 to 2019, among which nine lacked complete case information, 11 were lost to follow‐up, nine were not administered crizotinib, and 14 patients received crizotinib treatment without disease progression. Finally, 128 patients were included in the study (Figure 1). Among them, 12 (9.4%) were over 65 years of age, 67 (52.3%) were women, 33 (25.8%) had a smoking history, 50 (39.1%) had bone metastasis at baseline, 79 (61.7%) received at least one cycle of chemotherapy before crizotinib treatment, and 19 patients (14.8%) received three or more ALK‐TKI treatments (Table 1).

**TABLE 1:** Baseline characteristics

Characteristics	Overall patients (*n* = 128)
Age (y)	
Median (range)	51(23–77)
<65	116 (90.6)
≥65	12 (9.4)
Sex, *n* (%)	
Male	61 (47.7)
Female	67 (52.3)
Smoking history, *n* (%)	
Yes	33 (25.8)
No	95 (74.2)
ECOGPS, *n* (%)	
0	4 (3.2)
1	122 (95.2)
2	2 (1.6)
Pathological type, *n* (%)	
Adenocarcinoma	120 (93.7)
Non‐adenocarcinoma	8 (6.3)
Stage at diagnosis, *n* (%)	
III B	14 (10.9)
IV	114 (89.1)
Site of distant metastases, *n* (%)	
Bone	50 (39.1)
Brain	46 (35.9)
Liver	23 (18.0)
Adrenal gland	9 (7.0)
Number of distant metastases, *n* (%)	
<4	108 (84.4)
≥4	20 (15.6)
Testing method	
FISH	62 (48.5)
IHC	22 (17.2)
RT‐PCR	16 (12.5)
NGS	3 (2.3)
Unknown	25 (19.5)
ALK‐TKI as first‐line therapy, *n* (%)	
Yes	49 (38.3)
No	79 (61.7)
Number of ALK‐TKI, *n* (%)	
1	65 (50.8)
2	44 (34.4)
≥3	19 (14.8)

Abbreviations: y, years; ECOG‐PS, Eastern Cooperative Oncology Group‐performance status; FISH, fluorescence in situ hybridization; IHC, immunohistochemistry; RT‐PCR, real time‐polymerase chain reaction; NGS, next generation sequencing; ALK‐ TKI, anaplastic lymphoma kinase‐tyrosine kinase inhibitor.

### Clinical efficacy of enrolled patients

**FIGURE 2:**
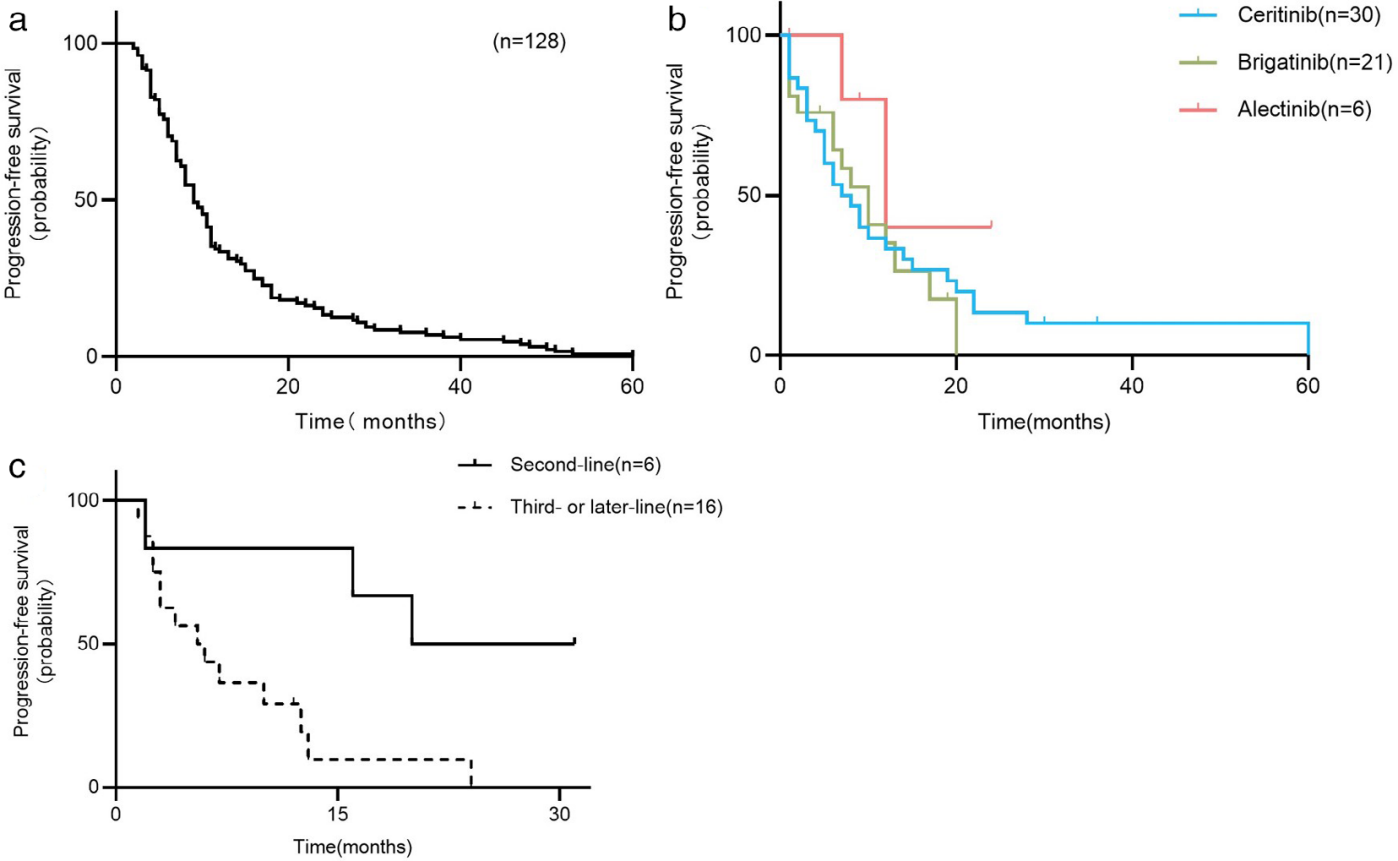
Kaplan–Meier curves for each ALK inhibitor (PFS). (a) The median PFS of crizotinib was 9.0 months (95% CI, 7.7–10.3 months). (b) The median PFS of ceritinib was 7.0 months (95% CI, 3.4–10.6 months); the median PFS of brigatinib was 10.0 months (95% CI, 6.1–13.9 months); the median PFS of alectinib was 12.0 months (95% CI, 4.7–19.3 months). (c) The median PFS of lorlatinib was 12.5 months (second‐line: 20.0 months vs. third‐ or later‐line: 5.5 months, *p* = 0.011). Abbreviations: ALK, anaplastic lymphoma kinase; PFS, progression‐free survival; CI, confidence interval; *p*‐values <0.05 were statistically significant

After 128 patients were assessed according to RECIST 1.1 evaluation criteria, the ORR of crizotinib was 68.0%, and DCR was 93.0%. By the last follow‐up, disease progression occurred in enrolled patients, and the median PFS (mPFS) of crizotinib was 9.0 months (95% confidence interval [CI], 7.7–10.3 months) **(**Figure 2(a)).

**FIGURE 3:**
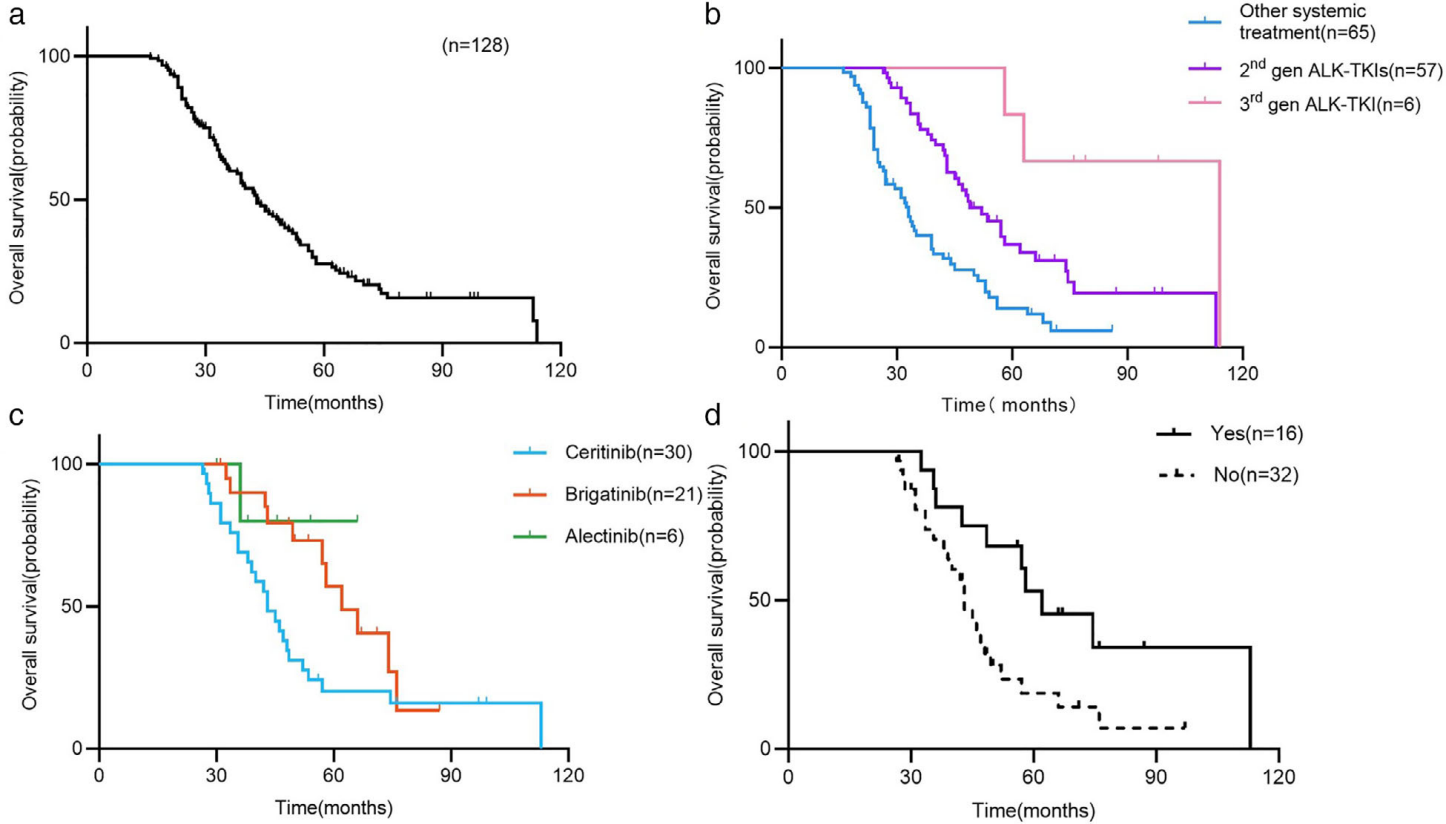
Kaplan–Meier curves for all patients and different sequential patterns after crizotinib progression (OS). (a) The median OS of 128 patients: 43.0 months (95% CI, 36.9–49.1 months). (b) Received other systemic treatment: 33.0 months (95% CI, 28.9–37.2 months); received the 2nd gen ALK‐TKI: 58.5 months (95% CI, 45.6–71.4 months); received the 3rd gen ALK‐TKI: 114.0 months (NR). (c) The median OS of ceritinib was 43.0 months (95% CI, 37.7–48.3 months); the median OS of brigatinib was 62.0 months (95% CI, 49.1–74.9 months); the median OS of alectinib was not reached. (d) The median OS with or without lorlatinib after first‐ and second‐generation drug resistance: 62.0 months versus 43.0 months, *p* = 0.014. Abbreviations: OS, overall survival; CI, confidence interval; 2nd gen, second generation; 3rd gen, third generation; ALK‐TKI, anaplastic lymphoma kinase‐tyrosine kinase inhibitor; NR, not reached; *p*‐values <0.05 were statistically significant

As per the last follow‐up date, 95 patients (74.2%) died. Overall, the median OS (mOS) was 43.0 months (95% CI, 36.9–49.1 months) (Figure 3(a**)**). Univariate analysis showed that patients age <65 years, without bone metastasis at baseline, and undergoing next‐generation *ALK* inhibitors had longer OS than other patients. Cox multivariate analysis showed that no smoking habit (hazard ratio [HR], 0.494; 95% CI, 0.258–0.946; *p* = 0.034), no bone metastasis (HR, 0.502; 95% CI, 0.295–0.854; *p* = 0.011), administration of second‐generation *ALK* inhibitors (HR, 0.584; 95% CI, 0.369–0.922; *p* = 0.021) and third‐generation *ALK* inhibitors (HR, 0.250; 95% CI, 0.115–0.547; *p* = 0.001) were related to longer OS (Table 2).

**TABLE 2:** Cox multivariate analysis of overall survival of all patients (*n* = 128)

Variables	Log‐rank test	Multivariate analysis		
		HR	95% CI	*p*‐Value
Sex (male vs. female)	0.850			
Age (≥65 y vs. <65 y)	0.037			
Smoking history (yes vs. no)	0.160	2.025	1.057–3.881	0.034
Adenocarcinoma (yes vs. no)	0.449			
Stage at diagnosis (IV vs. III)	0.169			
Number of distant metastases (≥4 vs. <4)	0.818			
Bone metastasis (yes vs. no)	0.019	1.994	1.171–3.394	0.011
Brain metastasis (yes vs. no)	0.214			
Liver metastasis (yes vs. no)	0.910			
Adrenal gland metastasis (yes vs. no)	0.093			
ALK‐TKI as first‐line therapy (yes vs. no)	0.090			
Received second‐generation ALK‐TKI (yes vs. no)	0.003	0.691	0.369–0.992	0.021
Received third‐generation ALK‐TKI (yes vs. no)	<0.001	0.250	0.115–0.547	0.001

Abbreviations: HR, hazard ratio; CI, confidence interval; *p*‐values <0.05 were statistically significant; ALK‐ TKI, anaplastic lymphoma kinase‐tyrosine kinase inhibitor.

### Efficacy of different sequential patterns in crizotinib‐resistant patients

#### Overall survival of different treatment patterns

In this study, 128 patients developed disease progression after crizotinib treatment, the mOS of 65 patients undergoing other systemic therapy was 33.0 months (95% CI, 28.9–37.2 months), and that of 57 patients administered sequential second‐generation ALK‐TKIs was 58.5 months (95% CI, 45.6–71.4 months). Further, mOS was 114.0 months (not reached [NR]) in six patients directly undergoing sequential third‐generation ALK‐TKI lorlatinib (Figure 3(b)).

Pairwise comparison between groups showed that the OS of sequential second‐generation ALK‐TKIs group was significantly longer than that of other systemic treatment group (*p* < 0.001); the OS of the directly sequential lorlatinib group was significantly longer than that of the sequential second‐generation ALK‐TKIs group (*p* = 0.020).

#### Differences in efficacy among second‐generation ALK‐TKIs

Fifty‐seven patients were sequentially treated with second‐generation ALK‐TKIs after crizotinib resistance. Among them, 30 ceritinib patients had the mPFS of 7.0 months (95% CI, 3.4–10.6 months) and mOS of 43.0 months (95% CI, 37.7–48.3 months). The mPFS and mOS of 21 patients with brigatinib were 10.0 months (95% CI, 6.1–13.9 months) and 62.0 months (95% CI, 49.1–74.9 months), respectively. The mPFS of six alectinib patients was 12.0 months (95% CI, 4.7–19.3 months) and mOS was not reached (only one patient died) (Figures 2(b) and 3(c)). Pairwise comparison between groups showed that OS of the sequential brigatinib group was significantly longer than that of the ceritinib group (*p* = 0.034), and there was no statistically significant difference among the other groups.

#### Differences in the efficacy of lorlatinib at different lines

**FIGURE 4:**
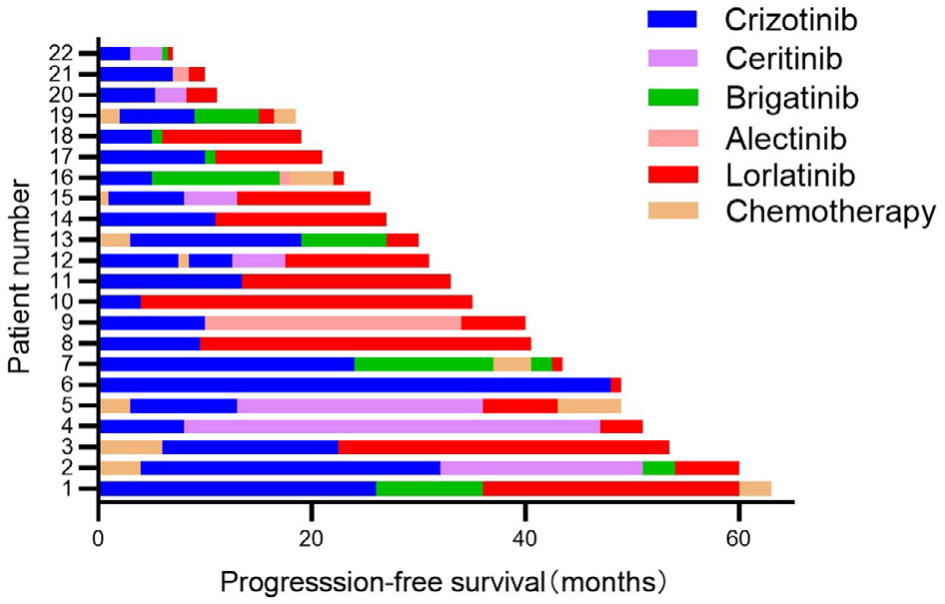
The specific treatment and progression‐free survival for patients who received lorlatinib.

Forty‐eight patients developed disease progression after first‐ and second‐generation ALK‐TKIs therapy, 16 of whom were sequentially treated with lorlatinib. Subgroup analysis showed statistically significant differences in OS with or without lorlatinib after first‐ and second‐generation drug resistance (62.0 months vs. 43.0 months, *p* = 0.014) (Figure 3(d)). Of the enrolled patients, 22 were administered lorlatinib. In addition to the 16 above‐mentioned patients, six patients were directly sequential with lorlatinib after crizotinib progression. Subgroup analysis showed that different lines of lorlatinib treatment had a significant effect on the PFS (second‐line: 20.0 months vs. third‐line or later: 5.5 months, *p* = 0.011) (Figure 2(c)). The specific treatment and PFS of patients who received lorlatinib are shown in Figure 4.

## DISCUSSION

To investigate the difference in survival of patients with advanced *ALK*‐positive NSCLC after crizotinib progression under different treatment patterns, we retrospectively analyzed the clinical data of 128 patients who received crizotinib as initial ALK‐TKI and demonstrated the clinical outcomes of different sequential patterns following crizotinib resistance.

Crizotinib was the only available *ALK* inhibitor before the second‐generation ALK‐TKIs was approved in China in December 2018; therefore, all patients in this study received crizotinib as the initial ALK‐TKI. Crizotinib was proven effective in the PROFILE series, and the mOS of 43.0 months in this study. Similarly, a previous study was reported the mOS of 48 months in the 121 patients treated with ALK‐TKIs.^13^, ^14^ Correlation analysis of clinical characteristics and efficacy of patients enrolled in the study showed that smoking history and bone metastasis at baseline had poor OS. The presence of brain and liver metastases at baseline and the number of metastases had no significant impact on OS, which was considered due partly to better control of multiple sites of metastases by the next‐generation of *ALK* inhibitors, and partly because of the small number of cases or local therapies. Ceritinib has a good effect on the liver, bone, and brain metastases in patients with resistance to crizotinib.^15^, ^16^, ^17^ Alectinib, brigatinib, and lorlatinib showed good intracranial response rates in the population treated with crizotinib.^10^, ^18^, ^19^, ^20^

After crizotinib resistance, survival was significantly better in 63 patients who were sequentially treated with other ALK‐TKIs than in 65 patients who were treated with other systemic therapies. Of these, the survival following treatment with second‐line directly sequential lorlatinib was significantly longer than with second‐generation *ALK* inhibitors. Previous real‐world analysis showed that the 5‐year OS rate of second‐line application of lorlatinib could reach 81.8%.^21^ Although there were only six patients administered lorlatinib after crizotinib in this study, the long‐term survival advantage of this prioritized lorlatinib pattern requires further investigation. Therefore, some randomized double‐blind clinical trials should be conducted analyzing ALK‐TKI administration after crizotinib resistance. Additionally, 16 of 48 patients with first‐and second‐generation *ALK* inhibitor progression had significantly longer survival with sequential lorlatinib. Some prospective studies showed good clinical efficacy of lorlatinib in second‐generation ALK‐TKIs resistant patients.^22^, ^23^, ^24^, ^25^ Previous real‐world studies in Japan (WJOG9516L) and France (IFCT‐1302 CLINALK) demonstrated the importance of sequential therapy.^26^, ^27^ However, the third‐generation ALK‐TKI still cannot overcome partial resistance mechanisms of second‐generation drugs. Therefore, more accurate genetic sequencing after ALK‐TKI resistance and development of new drugs against more resistance mechanisms are essential.

Until the next‐generation ALK‐TKI was recommended as a first‐line priority, sequential second‐generation *ALK* inhibitors following crizotinib failure was the standard mode of treatment. However, the efficacy of the sequential second‐generation *ALK* inhibitors varied between them. Randomized double‐blind trials comparing the efficacy of second‐generation ALK‐TKIs in crizotinib‐resistant patients with advanced *ALK*‐positive NSCLC are still lacking. Intergroup comparisons in this study showed that OS of the 21 sequential brigatinib patients was significantly longer than that of the 30 sequential ceritinib patients (intergroup comparisons with the alectinib group were not performed because only one patient died). However, a deeper look at the treatment after progression in the two groups showed that nine patients (42.9%) in the brigatinib group were subsequently treated with lorlatinib, compared with only five patients (16.7%) in the ceritinib group. Confounding factors for survival intervention cannot be completely excluded in retrospective analysis. Therefore, whether there is a difference in the survival following crizotinib resistance sequentially with different second‐generation ALK‐TKIs needs to be further verified by high‐standard clinical trials.

In recent years, the second‐generation ALK‐TKI has become the first‐line drug of choice for advanced *ALK*‐positive NSCLC. However, whether the PFS advantage of the next‐generation drugs can be translated into OS advantage remains to be verified. OS data from the recently published J‐Alex study in Japan showed no significant difference in 5‐year OS rates between patients receiving first‐line treatment with alectinib and crizotinib (60.85% vs. 64.11%).^28^ Unlike the ALEX study,^29^ the J‐Alex study allowed patients in the crizotinib group to switch to alectinib group before disease progression (such patients accounted for 78.8%), which was one of the reasons for the similar 5‐year OS rates of the two drugs. Further, the first‐line treatment with lorlatinib in the CROWN study yielded unexpected results.^30^

Our study has several limitations. This was a single‐center, retrospective study, with limited number of patients in each group and inevitable population bias. Additionally, the follow‐up time was not sufficiently long, and the follow‐up treatment and OS data of the enrolled patients need further improvement. However, dynamic circulating tumor DNA (ctDNA) detection of 35 patients receiving ALK‐TKIs of various generations is being conducted in our follow‐up study, which may provide further important information. The purpose of the follow‐up study is to further explore the correlation between genomic characteristics and the efficacy of ALK‐TKI before and after treatment and to clarify the mechanism of resistance to ALK‐TKIs among Chinese patients with advanced *ALK*‐positive NSCLC. Thereafter, a preliminary profile of genomic cloning evolution will be drawn.

In conclusion, through preliminary analysis of real‐world data, we increased our understanding of the clinical efficacy and factors influencing OS following administration of various generations of *ALK* inhibitors. The application of next‐generation ALK‐TKI after crizotinib failure significantly prolonged survival and direct sequencing lorlatinib seemed advantageous. Similarly, lorlatinib also prolonged survival in patients with first‐ and second‐generation ALK‐TKIs failure. The results of dynamic ctDNA molecular variation characteristics are expected to help develop precise treatments for advanced NSCLC.

## CONFLICT OF INTEREST

The authors declare that there are no conflicts of interest.
